# The Role of Roller Rotation Pattern in the Spreading Process of Polymer/Short-Fiber Composite Powder in Selective Laser Sintering

**DOI:** 10.3390/polym14122345

**Published:** 2022-06-09

**Authors:** Tan Cheng, Hui Chen, Qingsong Wei

**Affiliations:** 1 State Key Lab of Materials Forming and Die & Mould Technology, School of Materials Science and Engineering, Huazhong University of Science and Technology, Wuhan 430074, China; ctisvip@126.com; 2 Department of Mechanical Engineering, National University of Singapore, Singapore 117575, Singapore

**Keywords:** powder spreading, short fiber, packing quality, roller rotation pattern, discrete element method, selective laser sintering

## Abstract

In this study, for the first time, a forward-rotating roller is proposed for the spreading of CF/PA12 composite powder in the selective laser sintering (SLS) process. The mesoscopic kinetic mechanism of composite particle spreading is investigated by utilizing the “multi-spherical” element within the discrete element method (DEM). The commercial software EDEM and the open-source DEM particle simulation code LIGGGHTS-PUBLIC are used for the simulations in this work. It is found that the forward-rotating roller produces a strong compaction on the powder pile than does the conventional counter-rotating roller, thus increasing the coordination number and mass flow rate of the particle flow, which significantly improves the powder bed quality. In addition, the forward-rotating pattern generates a braking friction force on the particles in the opposite direction to their spread, which affects the particle dynamics and deposition process. Therefore, appropriately increasing the roller rotation speed to make this force comparable to the roller dragging force could result in faster deposition of the composite particles to form a stable powder bed. This mechanism allows the forward-rotating roller to maintain a good powder bed quality, even at a high spreading speed, thus providing greater potential for the industry to improve the spreading efficiency of the SLS process.

## 1. Introduction

Selective laser sintering (SLS), one of the popular processes for manufacturing polymer materials in additive manufacturing technology, has continued to receive wide attention from the community in recent years [1,2]. In the polymer additive manufacturing process, polymer/fiber composite powder materials have attracted a lot of interest from scholars because fibers can significantly enhance the mechanical properties of the matrix material [3,4].

The polymer/fiber composite SLS forming process consists of two essential parts. First, a roller is used to spread a certain thickness of powder particles on the forming substrate, and then a laser is applied to selectively sinter the powder [5,6]. Each of these aspects has a very important influence on the quality of the final formed part, and there has been a great deal of research related to the laser sintering process [7,8,9]. However, much less attention has been paid to the roller-type powder spreading process. In fact, during the powder spreading procedure, the final deposition quality of the powder bed, including the powder bed density and powder bed morphology, has a direct impact on the subsequent fabrication process [10,11]. For example, Haeri et al. [12] found that the shape of PEK HP3 particles spread in the powder bed had an effect on the subsequent laser sintering rate. Chen et al. [13] discovered that the anisotropy of carbon fibers (CF) caused by the CF/PA12 composite particles’ spreading process eventually led to the anisotropy of the sintered parts. All of these studies illustrate the importance of the powder spreading procedure for the SLS process. For polymer/fiber composite powder materials, the powder flowability is greatly decreased by the introduction of fibers, leading to a reduction in the quality of the powder bed [14,15]. Therefore, to obtain high-quality powder beds, it is necessary to study the effects of the powder spreading process on polymer/fiber composite materials’ powder bed quality and the related kinetic mechanism.

The loose nature of the particles in the powder bed makes it difficult to measure the quality parameters of the powder bed using in situ experimental methods [16,17], and the mesoscopic kinetic characteristics of the powder particles are almost impossible to observe directly through experiments [17,18]. Numerical simulation based on the discrete element method (DEM) is an effective way to study the characteristics of powder spreading [19,20]. Recently, many scholars have utilized the DEM method to investigate the factors affecting the quality of powder spreading by studying the roller-type spreading process. For example, Nan et al. [21] studied the effect of roller rotation speed on the quality of the spread powder bed via simulation, and they found that increasing the rotational speed caused size segregation of powder particles during the spreading process, thus reducing the uniformity of the powder bed. Zhang et al. [22] investigated the evolution of powder bed quality at different layer thicknesses, showing that the powder bed quality, including the relative packing density and surface uniformity, increased significantly with the increase in layer thickness. Chen et al. [23] studied the detailed dynamic mechanism of powder particles under different spreading speeds, including the powder velocity field, particle motion trajectory, and contact force distribution. They found that increasing the spreading speed of the roller increased the movement of the powder particles in the powder bed area, which was the essential reason for the deterioration of the powder bed quality. Meier et al. [24] simulated the effect of particle size distribution on the powder spreading process and found that a decrease in particle size increased the cohesion force between powder particles. Specifically, when the average particle size was reduced to 17 μm, the cohesion force increased to be two orders of magnitude larger than the particle’s gravity, leading to a large amount of powder agglomeration and thus a poorer packing quality. However, this existing research is based on the counter-rotating roller (i.e., the direction of linear velocity at the bottom of the roller is the same as the roller’s direction of movement), and little research has been conducted involving the forward-rotating roller (i.e., the direction of linear velocity at the bottom of the roller is opposite to the roller’s moving direction), where the powder spreading and deposition mechanisms are likely to be different.

In this study, for the first time, a method using the forward-rotating roller for polymer/short-fiber composite powder spreading is proposed. Polyamide12 (PA12), one of the most widely used polymer powders in SLS processes [5,25], and carbon fiber (CF), a commonly used fiber material [5], are selected for the composite powder spreading experiment. The DEM method is used to systematically study the kinetic mechanisms of the composite particles during the powder spreading process, using a forward-rotating roller, and a detailed comparison with the case of a counter-rotating roller is made. This provides a new approach for polymer/short-fiber composite powder spreading in SLS and other processes.

## 2. Numerical Model

### 2.1. Governing Equations

**Figure 1:**
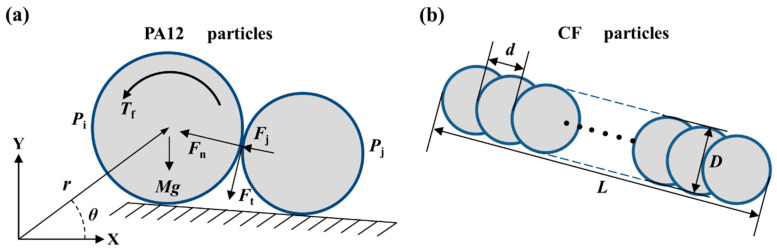
Schematic of the DEM particle model: (**a**) PA12 particles with “soft-spherical” element; (**b**) CF particles with “multi-spherical” element.

For spherical or near-spherical PA12 particles, the “soft-spherical” DEM model [26,27] was used to simulate the spreading process of the PA12 particles, a process which has been reported in our previous study; the applicability of the model to this process has been experimentally verified [14]. As shown in Figure 1a, a particle i in the particle flow is represented by a spherical unit. The normal force *F*_n_, and tangential force *F*_t_, generated by contact with the neighboring particle j, were calculated using the Hertz–Mindlin model [28], given as:

(1) $$F_{n}=-\sqrt{\frac{20}{3}}\varphi {\left[E^{*}M^{*}{\left(R^{*}S_{n}\right)}^{\frac{1}{2}}\right]}^{\frac{1}{2}}\dot{S_{n}}+\frac{4}{3}E^{*}R^{*\frac{1}{2}}S_{n}^{\frac{3}{2}}\;$$

(2) $$F_{t}=-min\left\{\mu_{s}F_{n},\left|\sqrt{\frac{80}{3}}\varphi {\left[G^{*}M^{*}{\left(R^{*}S_{n}\right)}^{\frac{1}{2}}\right]}^{\frac{1}{2}}\dot{S_{t}}+8G^{*}R^{*\frac{1}{2}}S_{n}^{\frac{1}{2}}S_{t}\right|\right\}$$

where *µ*_s_ is the sliding friction coefficient; *S*_n_ (µm) and *S*_t_ (µm) are the normal and tangential relative displacements between particle i and particle j, respectively. Note that the transform coefficient *φ* and the equivalent physical properties, i.e., particle mass M* (g), particle radius R* (µm), Young’s modulus E* (Gpa) and shearing modulus G* (Gpa), are respectively defined as:

(3) $$\;\{\begin{array}{c} \varphi =\frac{\ln e}{{\left(\ln^{2}e+\pi^{2}\right)}^{\frac{1}{2}}}\\ \;M^{*}=\frac{M_{i}M_{j}}{M_{i}+M_{j}}\\ R^{*}=\frac{R_{i}R_{j}}{R_{i}+R_{j}}\\ E^{*}=\frac{E_{i}E_{j}}{\left(1-\gamma_{j}^{2}\right)E_{i}+\left(1-\gamma_{i}^{2}\right)E_{j}}\\ G^{*}=\frac{G_{i}G_{j}}{\left(1-\gamma_{j}\right)G_{i}+\left(1-\gamma_{i}\right)G_{j}} \end{array}$$

where *M*_i_ and *M*_j_, *R*_i_ and *R*_j_, *E*_i_ and *E*_j_, *G*_i_ and *G*_j_, and *γ*_i_ and *γ*_j_ represent the mass, radius, Young′s modulus, shear modulus, and Poisson′s ratio of particle i and particle j, respectively; *e* is the restitution coefficient between the two particles. 

For granular flow systems, cohesion forces, such as the liquid bridge force, electrostatic force, and van der Waals forces, have significant effects on particle dynamics. However, in the SLS process, the composite particles need to be dried in a vacuum environment before the powder spreading and laser sintering procedures, thus the effect of the liquid bridge force is negligible. In addition, the CF particles have strong electrical conductivity, so the role of the electrostatic force in composite particles should also be small and can be disregarded. What needs to be incorporated into the model are the van der Waals forces, as the composite particles are only tens of microns in size. According to the JKR cohesion theory [29], the cohesion force between particles caused by the van der Waals forces can be given by the following equation:

(4) $$F_{J}=-{\left(\frac{8\pi WE^{*}a^{3}}{1-\gamma^{2}}\right)}^{\frac{1}{2}}$$

where *W* (mJ/m^2^) is the surface energy density of the particle; *a* (µm) is the contact radius between the two particles.

For the non-spherical CF particles with large aspect ratios, the “multi-spherical” element method [30] was used for modeling, an approach which has also been validated in our previous work [14]. As shown in Figure 1b, the CF particles were composed of a set of parallel spherical units that partially overlap with each other. For a given length of CF particles, the more overlap between adjacent spheres (i.e., the smaller the sphere center distance *d*), the more accurately the fibers can be modeled. However, this leads to a significant increase in computational time costs. In this paper, the center distance of the spheres was fixed at *D*/2. Thus, the CF particles of different lengths were modeled by increasing or decreasing the number of spheres. The total length *L* of the overlapping spheres was the length of the CF particles, and the diameter *D* of the sphere units was the end face diameter of the particles. The mass and rotational inertia of the CF particles can be calculated by Boolean subtraction. Note that, considering the short length and high strength of CF particles, it was assumed that the fibers would not break and bend during the spreading process.

Since the CF particles were modeled by spherical units, the contact detection law and the constitutive equation of interparticle forces for the “soft-spherical” element were still available for the mixtures of CF and PA12 powder; thus, Equations (1)–(3) could be used for PA12/CF composite particles. As such, the translation, as well as the rotation, of the composite particles during the spreading process is defined as:

(5) $$M_{i}\frac{d^{2}r_{i}}{dt^{2}}=M_{i}g+\sum_{j}\left(F_{n}+F_{t}+F_{J}\right)\;\;$$

(6) $$I_{i}\frac{d^{2}\theta_{i}}{dt^{2}}=-\mu_{r}F_{n}R_{i}\frac{{\dot{\theta }}_{i}}{\left|{\dot{\theta }}_{i}\right|}+\sum_{j}\left(F_{t}\times R_{i}\right)\;$$

where *r*_i_ (µm), *θ*_i_ (rad), and *I*_i_ (kg·m²) are the displacement, angular displacement, and rotational inertia of the particle i, respectively; *µ*_r_ is the rolling friction coefficient; and *g* (m/s^2^) is the gravity acceleration.

### 2.2. Time Step

Before performing the DEM simulation, a suitable time step needed to be determined to balance the simulation time and calculation accuracy. In this study, the time step used to calculate the incremental forces and displacements of the composite particles was determined by the Rayleigh critical time step [31] Δ*t*:

(7) $$\Delta t=\pi {\left[\frac{R}{0.163\gamma +0.877}\sqrt{\frac{\rho }{G}}\right]}_{\min}$$

where *R* (µm), *ρ* (g/cm^3^), *γ*, *G* (Gpa) are the radius, density, Poisson′s ratio, and shear modulus of the composite particles, respectively.

### 2.3. Simulation Parameters and Conditions

The DEM parameters of the composite particles used in the simulations are listed in Table 1. The friction coefficient between the composite particles was measured using the repose-angle-testing method [32]. Considering that the surface of the sandblasted substrate is very rough in the actual SLS process, the friction coefficient of the particle-substrate was thus set to double that of the corresponding particle–particle friction coefficient. In addition, the friction coefficient of the particle-roller was set to half the value of the corresponding particle–particle friction coefficient owing to the smooth surface of the roller. The Young′s modulus of the composite particles was two orders of magnitude smaller than that of the actual material, which effectively increased the Rayleigh critical time step and thus made the computation cost tolerable. Then, the surface energy density of the powder was correspondingly reduced by two orders of magnitude, rendering the ratio between Young′s modulus and Hamaker′s constant unchanged and thus allowing for a more accurate calculation of the van der Waals forces, which has been discussed extensively in the existing literature [17,33].

**Figure 2:**
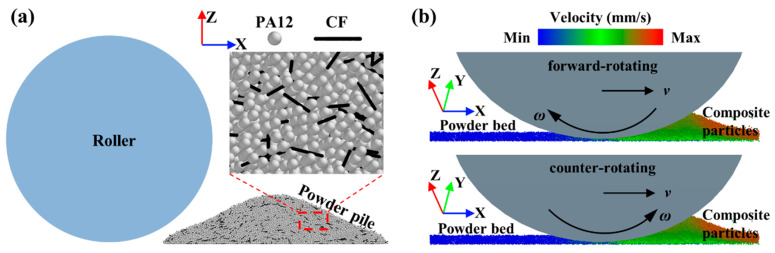
Composite particle spreading process: (**a**) generated PA12/CF composite particles; (**b**) composite particle spreading with different roller rotation patterns.

The mixture of PA12 and CF particles, modeled with the “soft-spherical” element and “multi-spherical” element methods, is shown in Figure 2a. Before the powder spreading, the composite particles of random size and orientation were uniformly generated in the space above the substrate and subsequently fell onto the substrate to form a powder pile, where the mass ratio of PA12 particles to CF particles was 7:3. The diameter and length distribution of CF powder were 10 μm and 40–180 μm, respectively, and the particle size distribution of PA12 powder was 30–70 μm. All the physical parameters used in the model for the mixed powder are similar to those of the particles used in our previous study, and they have been experimentally verified [14]. Different patterns of rollers (i.e., forward-rotating, counter-rotating, and non-rotating) were used to simulate the powder spreading process, as shown in Figure 2b, where the roller diameter *D*_r_ and the layer thickness *h* were fixed at 10 µm and 100 µm, respectively; the spreading speed *v* and the roller rotation speed *ω* were 50–150 mm/s and 0–4.8π rad/s, respectively. Note that the calculation domain along the X direction, i.e., the spreading direction, was 100 mm, which is comparable to the actual process. Furthermore, the calculation domain along the Y direction was set to 0.5 mm, with a periodic boundary to reduce the calculation cost. The commercial software EDEM (EDEM2018, DEM-Solutions, Ltd., Edinburgh, UK) and the open-source DEM particle simulation code LIGGGHTS-PUBLIC [34] were used for the simulations in this study. The computation time for one simulation case on our desktop workstations with the Intel Xeon W-2145 CPU was about 140 h. 

Finally, after the simulation of a powder spreading procedure, the relative packing density *ρ*_d_ of the powder bed was determined by the following equation:

(8) $$\rho_{d}=\frac{M_{b}}{Sh\left(\rho_{1}\times 70\%+\rho_{2}\times 30\%\right)}\times 100\%\;$$

where *M*_b_ (g) and *S* (mm^2^) are the mass and area of the spread powder bed, respectively; *ρ*_1_ (g/cm^3^) and *ρ*_2_ (g/cm^3^) are the densities of PA12 and CF, respectively.

**Table 1:** Processing parameters employed in the simulations.

Parameters	Symbols	Values		
		PA12	CF	PA12-CF
Density	*ρ* (g/cm^3^)	1.76	1.01	-
Young′s modulus [35,36]	*E* (Gpa)	230	2.3	-
Poisson′s ratio [35,36]	*γ*	0.3	0.4	-
Sliding friction coefficient [13]	*µ*_s_	0.45	0.3	0.4
Rolling friction coefficient [13]	*µ*_r_	0.01	0.01	0.01
Restitution coefficient [13]	*e*	0.5	0.8	-
Surface energy density [35,36]	*W* (mJ/m^2^)	32	38	-
Spreading speed	*v* (mm/s)	50–150		
Rotation speed	*ω* (rad/s)	0–4.8π		
Layer thickness	*h* (µm)	100		
Roller diameter	*D*_r_ (mm)	10		
Particle size distribution	*λ*_1_ (µm)	30–70		
Fiber length distribution	*λ*_2_ (µm)	40–180		
Fiber diameter	*D*_f_ (µm)	10		

## 3. Results and Discussion

### 3.1. Packing Quality of Powder Bed

**Figure 3:**
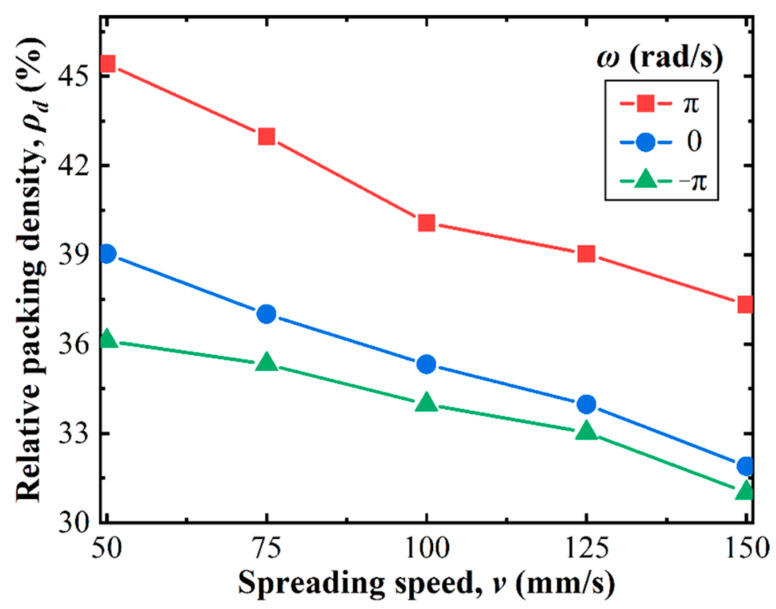
Evolutions of the powder bed packing density under different roller rotation patterns.

Figure 3 illustrates the comparison of the relative packing density of the powder bed ($\rho_{d}$) for three different roller rotation patterns, where the spreading speed is from 50 mm/s to 150 mm/s. As shown, the packing density of the powder bed decreased monotonically with increased spreading speeds for different rotation patterns, a phenomenon analogous to those reported in many previous studies [23,37]. However, for a given spreading speed, the packing density with the forward-rotating roller was significantly better than the other two patterns.

**Figure 4:**
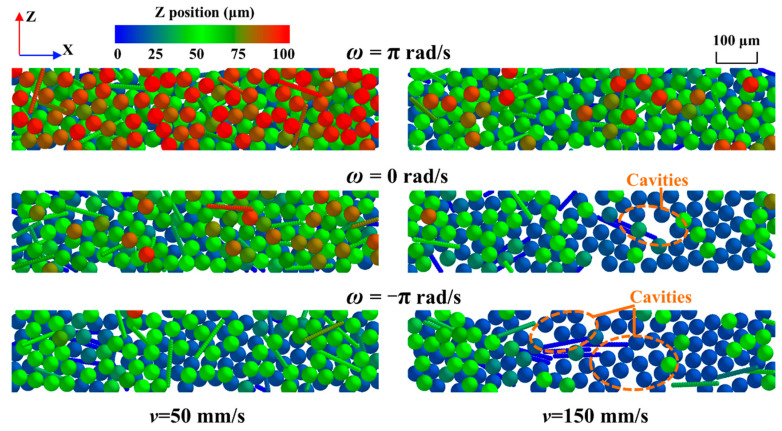
Surface morphology of the powder bed under different roller rotation patterns.

The surface morphology of the powder bed under different roller rotation patterns is shown in Figure 4, where the powder bed profile is colored by the height value in the Z-direction. When the forward-rotating roller was used, as shown, the powder bed became thicker and smoother with fewer voids (i.e., the color of the powder bed becomes redder), meaning that more CF and PA12 powder particles were spread into the powder bed, thus increasing its density.

**Figure 5:**
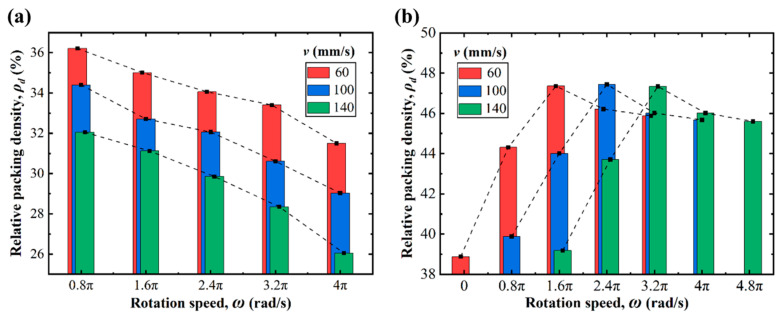
Evolutions of packing density with different rotation speeds and spreading speeds in a (**a**) counter-rotating pattern and a (**b**) forward-rotating pattern.

Figure 5 depicts the evolution of powder bed packing density with different rotational speeds (*ω*) and spreading speeds (*v*) when the forward-rotating and counter-rotating rolls were used, respectively. For the counter-rotating roller, the powder bed packing density decreased monotonically with increases to the rotational speed, which indicates that increasing the rotational speed is detrimental to the spreading process. In contrast, for the forward-rotating roller, as the rotation speed increased, the packing density first increased to a certain level and then started to decrease. This indicates that there is an optimal rotational speed when the forward-rotating roller is applied. Furthermore, this optimal rotational speed increased gradually with increasing spreading speed, and the powder bed quality obtained at the optimal rotational speed was essentially the same.

From the above numerical simulation results, it can be seen that the forward-rotating pattern can significantly improve the powder bed quality for the same process parameters. In addition, for the conventional counter-rotating roller, the processing efficiency and the powder bed quality cannot be combined [23]; i.e., the spreading speed must be sacrificed if the packing quality is to be improved. However, for the forward-rotating mode with a high spreading speed, the packing quality of the powder bed can be improved by increasing the rotation speed appropriately. This means that, by using a forward-rotating roller with an optimal combination of rotation speed and spreading speed, processing efficiency can be improved while a high-quality powder bed is maintained. The reasons why the forward-rotating pattern is better than the counter-rotating pattern is discussed in detail in Section 3.2. The optimal rotation speed for the forward-rotating pattern is discussed in Section 3.3.

### 3.2. Kinetic Comparison of Composite Powder Particles in Forward-Rotating and Counter-Rotating Patterns

**Figure 6:**
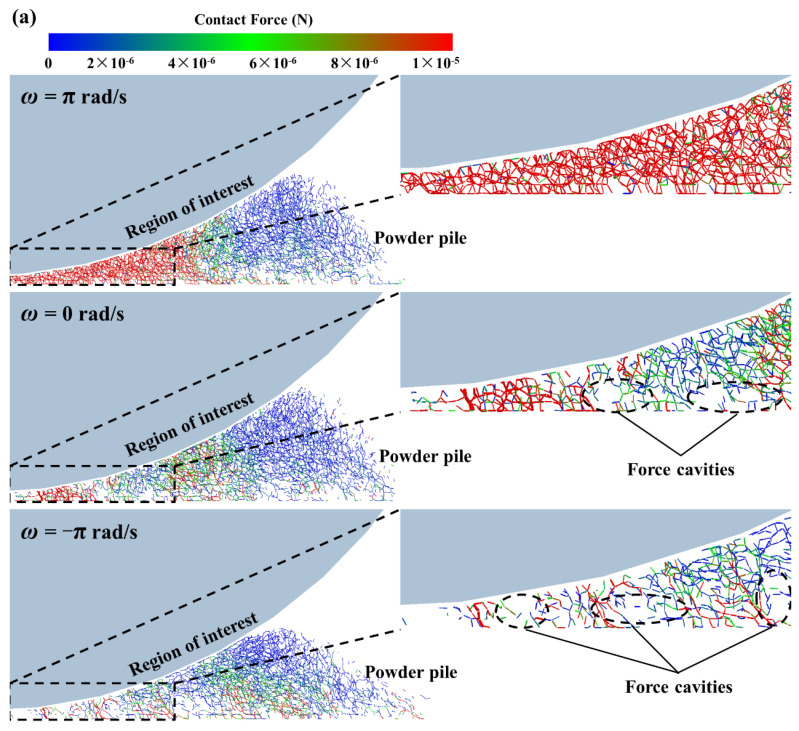

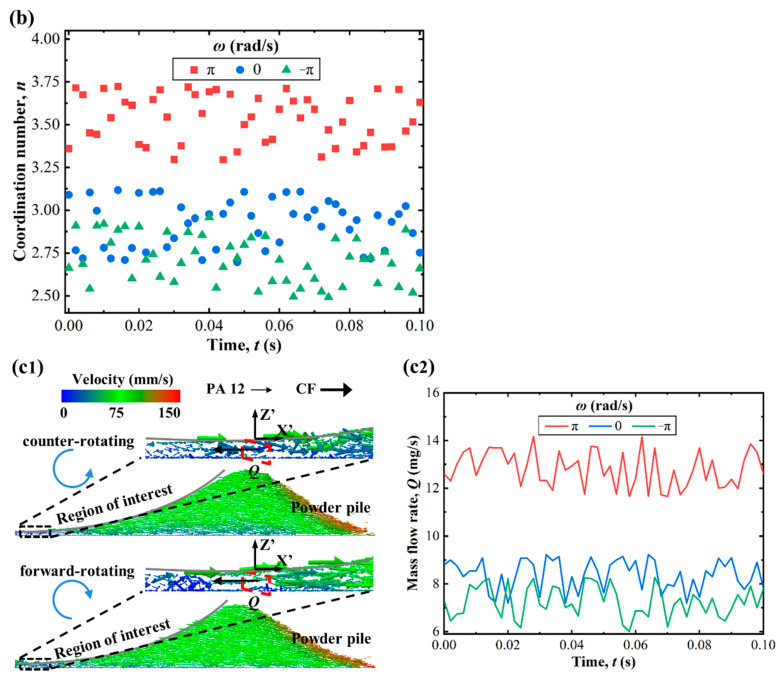
DEM simulation results in different rotation modes: (**a**) contact force between composite powder; (**b**) evolution of the coordination number; (**c1**) velocity field of the composite particles and (**c2**) mass flow rate.

The dynamics of the composite particles directly affect the particle spreading and deposition processes and, hence, the final powder bed packing quality, which is illustrated in detail in the DEM simulations in Figure 6. Figure 6a shows the contact force network distribution in the powder pile during the spreading process for three different rotation patterns, where the spreading speed is 100 mm/s. Note that the contact forces between the particles are represented by the small sticks in the figure, and their magnitude is indicated by the color scale bars. We focused on the powder in the “region of interest”, i.e., the powder in the gap between the bottom of the roll and the substrate (shown in the black dashed box in the figure), which was deposited onto the substrate to form the powder bed. As shown in the enlarged image in the upper right corner, for the counter-rotating pattern (or the non-rotating pattern), the contact force between the composite particles was weak, with more force cavities in the powder pile. This indicates that the roller compaction of the powder pile was relatively weak, thus forming a looser powder flow, which was detrimental to the powder spreading. However, when the forward-rotating mode was used, the roller produced a strong compaction of the powder pile, resulting in a significant reduction in force cavities in the powder pile and, hence, facilitating a denser powder bed formation.

The coordination number (*n*) between powder particles is an indicator that characterizes the degree of the powder bed’s compaction. In order to quantify the powder bed’s compaction under different rotational modes, the evolution of the coordination number in the “region of interest” during the spreading process is plotted in Figure 6b. The coordination number is larger in the forward-rotating mode than in the other two modes, which means that the number of contacts between the composite particles was higher when spreading powder with the forward-rotating roller, and, thus, a denser powder bed was obtained.

Figure 6c shows the particle velocity field along the spreading direction for different rotational modes. We focused on powder movement in the “region of interest” and monitored the mass flow rate (*Q*) of the particles at the gap between the bottom of the roller and the substrate (the red dashed area in the figure). Compared to the counter-rotating pattern (or the non-rotating pattern), the mass flow rate was consistently higher in the forward-rotating mode, indicating that more powder flow entered the gap region, thus forming a denser powder bed.

In summary, when the composite powder is spread by the forward-rotating pattern, the roller can produce a strong compaction on the powder pile, increasing the coordination number and mass flow rate of the particle flow, thus allowing more particles to be deposited onto the substrate, thereby forming a denser powder bed. This is the reason why the forward-rotating pattern is superior to the traditional, counter-rotating mode.

### 3.3. Effects of Rotation Speed

**Figure 7:**
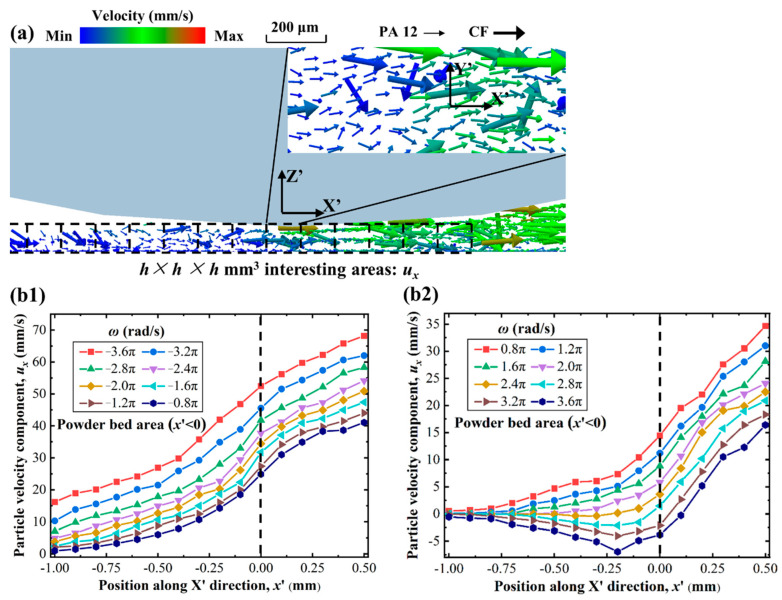

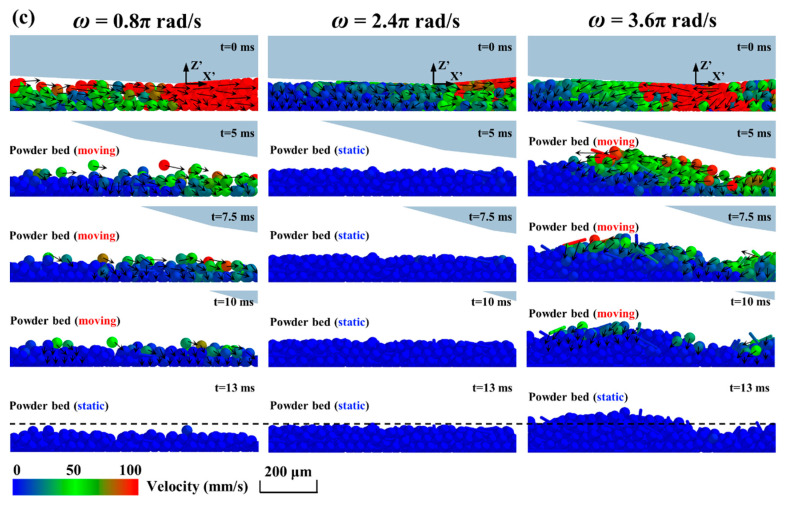
DEM results showing the composite powder motion and deposition processes: (**a**) example of particle velocity profile along the “interesting areas”; particle velocity profiles at different rotation speeds from (**b1**) counter-rotating mode to (**b2**) forward-rotating mode; (**c**) detailed composite particle deposition process at different rotation speeds in forward-rotating mode.

The rotation speed of the roller affects the dynamics of the composite particles and, therefore, changes the deposition process of the particles. To investigate the effect of rotation speed on the deposition procedure of the particles, a coordinate system of X′Y′Z′ that could move with the roller was established at the roller bottom, and a series of *h* × *h* × *h* mm^3^ “interesting areas” were set up in the gap region to monitor the particles’ motion and deposition process, as shown in Figure 7a. In the process of powder spreading, the particles move mainly along the spreading direction (i.e., the X′ direction) under the dragging force of the roller, while the motion along the Y′ direction is weak and negligible, as shown in the enlarged image in the upper right corner. Therefore, we concentrated on the movement of the particles along the X′ direction.

Figure 7b1 plots the particle velocity component (*u*_x_) profile along the “interesting areas” at different rotation speeds in the counter-rotating mode, where the spreading speed is 100 mm/s. The particle velocity in the powder bed area (i.e., *x*′ < 0) remained positive, which means that when the composite particles were discarded by the roller, they continued their motion along the spreading direction rather than immediately depositing onto the substrate. This particle movement is detrimental to the formation of a denser powder bed. Furthermore, the higher the rotation speed of the counter-rotating mode, the stronger the movement of the particles, which results in a lower quality of powder bed.

The situation was different when the forward-rotating mode was applied, as shown in Figure 7b2. With increases in rotational speeds, particle velocity in the powder bed region gradually decreased from positive to negative. Specifically, when the rotation speed reached 2.4π rad/s, the particle velocity was at a minimum, which means that the particles deposited onto the substrate more quickly and formed a stable powder bed. The reason for this phenomenon is that when the roller is rotating forwards, it generates a braking friction force that moves in the opposite direction to the spreading direction on the powder particles, which increases gradually with the increase in rotational speed. When this force is comparable to the dragging force of the roller, the combined force on the particles along the spreading direction is the smallest, thus making it easiest for the particles to be stationary.

The effect of braking friction on the composite powder deposition process can be verified in Figure 7c. When the rotational speed of the forward-rotating roller was slower (i.e., *ω* = 0.8π rad/s), the braking friction was weaker than the dragging force, causing the particles to continue moving forward after leaving the roller. On the contrary, when the rotational speed was faster (i.e., *ω* = 3.6π rad/s), the braking friction was stronger, causing the powder to move backward and form a local accumulation in the powder bed area. Neither set of conditions are favorable for particle deposition, which makes the particles stop moving until about t = 13 ms after entering the powder bed region. Significantly, at the optimal rotational speed (i.e., *ω* = 2.4π rad/s), the particles are completely stationary after only 5 ms, thus forming a high-quality powder bed. This is the reason why this optimal rotational speed exists in the forward-rotating mode.

From the above results and analysis, it can be concluded that the forward-rotating roller is superior to the conventional, counter-rotating roller. In industrial applications, by using a forward-rotating roller with an optimal combination of rotational speed and spreading speed, processing efficiency can be improved while a high-quality powder bed is maintained. This provides more potential options for enhancing the quality of the powder bed in SLS and other powder bed additive manufacturing processes.

## 4. Conclusions

In this study, for the first time, the forward-rotating roller was proposed for polymer/fiber composite powder spreading in the SLS process, and it was compared to the conventional, counter-rotating mode. The dynamics of composite particles during the spreading process in different roller rotation modes were investigated using the DEM numerical method, where the composite particles were modeled by “soft-spherical” and “multi-spherical” elements. Some of the main points and conclusions are highlighted below:

(1) Under the same process parameters, the forward-rotating roller produced stronger compaction on the composite powder pile, thus increasing the coordination number and mass flow rate of the particle flow. Therefore, the forward-rotating mode significantly improved the packing density and surface morphology of the powder bed compared to the counter-rotating and non-rotating modes.

(2) For the counter-rotating mode, when the rotation speed increased, the motion of the composite particles along the spreading direction (i.e., X direction) monotonically increased; this particle motion is detrimental to the formation of a stable powder bed. Consequently, it is ill-advised to increase the rotation speed of the counter-rotating mode.

(3) The forward-rotating roller exerted a braking friction force on the composite particles in the opposite direction to their spread, which increased with the increase in rotational speed. When this force reached a level comparable to that of the dragging force of the roller (i.e., when the rotational speed increased to a suitable level), the particles were deposited more quickly onto the substrate to form a dense powder bed. Thus, the forward-rotation roller has the optimal combination of rotation speed and spreading speed.

## Data Availability

Not applicable.
